# Heavily Graphitic-Nitrogen Self-doped High-porosity Carbon for the Electrocatalysis of Oxygen Reduction Reaction

**DOI:** 10.1186/s11671-017-2364-6

**Published:** 2017-11-17

**Authors:** Tong Feng, Wenli Liao, Zhongbin Li, Lingtao Sun, Dongping Shi, Chaozhong Guo, Yu Huang, Yi Wang, Jing Cheng, Yanrong Li, Qizhi Diao

**Affiliations:** 1 Research Institute for New Materials Technology, School of Chemistry and Chemical Engineering, Engineering Research Center of New Energy Storage Devices and Applications, Chongqing University of Arts and Sciences, Chongqing, 402160 China; 2 Central Laboratory Yongchuan Hospital, Chongqing Medical University, Chongqing, 402160 China

**Keywords:** Kidney bean, High porosity, Carbon material, Electrocatalyst, Oxygen reduction reaction

## Abstract

Large-scale production of active and stable porous carbon catalysts for oxygen reduction reaction (ORR) from protein-rich biomass became a hot topic in fuel cell technology. Here, we report a facile strategy for synthesis of nitrogen-doped porous nanocarbons by means of a simple two-step pyrolysis process combined with the activation of zinc chloride and acid-treatment process, in which kidney bean via low-temperature carbonization was preferentially adopted as the only carbon-nitrogen sources. The results show that this carbon material exhibits excellent ORR electrocatalytic activity, and higher durability and methanol-tolerant property compared to the state-of-the-art Pt/C catalyst for the ORR, which can be mainly attributed to high graphitic-nitrogen content, high specific surface area, and porous characteristics. Our results can encourage the synthesis of high-performance carbon-based ORR electrocatalysts derived from widely-existed natural biomass.

**Electronic supplementary material:**

The online version of this article (doi:10.1186/s11671-017-2364-6) contains supplementary material, which is available to authorized users.

## Background

Platinum (Pt)-based materials, the state-of-the-art catalysts for fuel cells, suffer from expensive price, limited resources, insufficient durability, and methanol-tolerant property in electrocatalysis process of oxygen reduction reaction (ORR) [1]. Great efforts were recently devoted to search for highly active, durable, and inexpensive alternatives to Pt-based ORR electrocatalysts for this purpose [2]. Among the various non-Pt catalysts, heteroatom-doped porous carbons (HDPC) are a new type of metal-free catalysts with high activity and durability for ORR thanks to their low-cost, non-toxicity, and renewability [3–6], and thus, the in-depth researches are eagerly anticipated to date. HDPC is generally synthesized by chemical methods or natural templates, but they cannot meet the requirements of low-cost, easy-to-synthesis, and excellent performance [7, 8]. Therefore, the search for a reasonable and effective method to synthesize the HDPC material is still a significant scientific issue for realizing highly-efficient catalysis for oxygen reduction.

As previously reported, protein-enriched biomass (e.g., nori [9], sweet potato vine [10], pomelo peel [11], enoki mushroom [12], coprinus comatus [13], and *Lemna minor* [14]) can be widely used a single-source precursor for HDPC catalyst towards the ORR. We recently propose some strategy to form the HDPC catalyst with porous 3D-network structure via high-temperature carbonization of fish-scale biowaste with an activator of zinc chloride [6]. We interestingly find that the first-step pretreatment of biomass can not only help to improve the characteristics of carbon structure of final ORR catalyst, but also increases its surface nitrogen content and doping efficiency of nitrogen atoms into carbon structure. Based on this finding, here, we first report a strategy to fabricate heavily graphitic-nitrogen-doped porous carbons (KB350Z-900) by directly converting white kidney bean (KB) biomass with a process of two-step carbonization, followed by zinc chloride activation, and acidic-treatment process. The KB biomass, which is one of the most famous edible beans today, can be abundantly and cheaply obtained in various countries. The total content of biological protein in dehydrated KB biomass is 20–30% generally. To the best of our knowledge, there is seldom reported on the ORR activity of the doped carbon catalyst derived from KB biomass. The role of ZnCl_2_ in the activation process can mainly spur the rapid dehydration and catalytic dehydroxylation of KB biomass so that the hydrogen and oxygen inside the KB biomass are released in the formation of water vapor. This process can facilitate the formation of more micro/meso-pores, finally producing nitrogen self-doped high-porosity carbon materials. The obtained carbon-based catalyst exhibits high electrocatalytic activity, long-term durability, and methanol-tolerant property, which may be a promising alternative to the Pt-based catalyst towards the ORR in alkaline medium.

## Methods

First, white kidney bean (KB) was washed by deionized water and completely dried at 80 °C in a vacuum drying oven. Subsequently, KB was pretreated in flowing-N_2_ atmosphere at 350 °C for 2 h for effective decomposition of protein to yield the KB350 precursor. Although the fastest decomposition of white KB biomass occurs at about 300 °C (Additional file 1: Figure S1), but a temperature of 350 °C was chosen as the first-step carbonization temperature in order to exceed the decomposition temperature of tyrosine (344 °C), the highest among the amino acids in bioprotein. KB350 and zinc chloride (ZnCl_2_) were mechanically mixed by ball-milling at 500 rpm according to mass ratio of 1:1. The obtained mixture was pyrolyzed in a tubular furnace at different temperatures (700, 800, 900, or 1000 °C) for 2 h with a heating-rate of 10 °C min^− 1^. The produced nanocarbon is hereafter called KB350Z-X (X = 700, 800, 900, or 1000). As a control, the KB-Z-900 was similarly fabricated by pyrolyzing a mechanical mixture of KB and ZnCl_2_ with the same mass ratio. Direct pyrolysis of KB at 900 °C for 2 h was utilized to prepare the KB900. All prepared samples were further post-treated in 0.5 mol l^− 1^ HCl solution for 2 h. The aim of acid-leaching is to effectively remove Zn species and metallic impurities before electrochemical testing.

Raman spectroscopy data were tested with a Renishaw inVia unit with an excited-λ of 514.5 nm. Field-emission scanning electron microscopy (FE-SEM) images were obtained by Hitachi UHR S4800 (Japan). High-resolution transmission electron microscopy (HR-TEM) was carried out on FEI Tecnai F30 instrument and acceleration voltage is 300 kV. X-ray photoelectron spectroscopy (XPS) was carried out using a Kratos XSAM800 spectrometer. A Micromeritics Analyzer (ASAP 2010) was applied to measure N_2_ adsorption/desorption isotherms at 77 K.

Electrochemical measurements were performed on a Zennium-E workstation (Germany) with a conventional three-electrode system. A glass-carbon rotation disk electrode (GC-RDE, Φ = 4 mm, Model 636-PAR), a saturated calomel electrode (SCE), and a graphite rod (Φ = 0.5 cm) were used as working electrode, reference electrode, and auxiliary electrode, respectively. The fabrication of working electrode refers to our previous reports [6]. Generally, 5.0 μl of 10 mg ml^− 1^ dispersion was transferred onto the GC-RDE surface and dried naturally. The mass loading of all tested samples was controlled to be ~ 400 μg cm^− 2^. All potentials (versus SCE) were transformed into the potentials versus the reversible hydrogen electrode (RHE).

## Results and Discussion

**Fig. 1:**
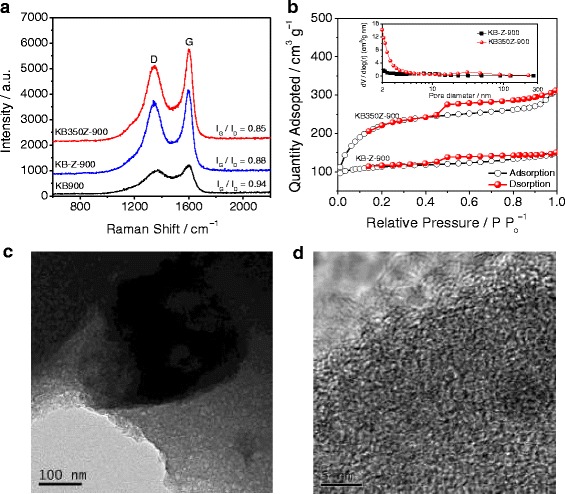
**a** Raman spectra of KB900, KB-Z-900 and KB350Z-900. **b** Nitrogen adsorption-desorption isotherms and corresponding BJH pore-size distributions (inset) of KB-Z-900 and KB350Z-900. **c** Low-resolution and **d** high-resolution transmission electron microscopy images of KB350Z-900

We have first tested the Raman spectra of KB900, KB-Z-900, and KB350Z-900 catalysts to understand their differences of structural properties. The Raman spectra are shown in Fig. 1a. The intensity ratio (I_D_/I_G_) of “D” band to “G” band was used to characterize the disordered and graphitic degrees. The I_D_/I_G_ is 0.85 for KB350Z-900 only, but the I_D_/I_G_ is 0.94 for KB900 and 0.88 for KB-Z-900, respectively. It may show that higher graphitic degree can be obtained at KB350Z-900 compared with as-prepared nitrogen/carbon (NC)-based catalysts, which can be directly confirmed by comparison of Raman intensity. Besides, the use of ZnCl_2_ activator in synthesis of NC-based catalysts can facilitate the enhancement of the graphitic degree during pyrolysis process owing to a lowest I_D_/I_G_ ratio of KB350Z-900. The first-step pretreatment of KB at 350 °C can further improve the graphitic degree of NC-based catalysts, which can help to produce more graphitic-nitrogen-doped carbon structures. N_2_ adsorption-desorption isotherms were used to investigate the effects of ZnCl_2_ activator and first-step pretreatment on the specific surface area and pore distribution of NC-based catalysts. Figure 1b clearly exhibits a Langmuir IV isotherm curve with a type H2 hysteresis loop, demonstrating that the mesoporous structures are also included in the prepared catalysts (e.g., KB-Z-900 and KB350Z-900). The BET-specific surface area is about 380 m^2^ g^− 1^ for KB-Z-900 and 1132 m^2^ g^− 1^ for KB350Z-900, respectively. A higher total pore volume of KB350Z-900 is ~ 0.62 m^3^ g^− 1^, and the meso- and macropore area of KB350Z-900 is ~ 664 m^2^ g^− 1^ (inset of Fig. 1b). First-step pretreatment of KB at 350 °C can promote the formation of more meso- and macropores and the increasing of BET specific surface area, further making for the exposure of active sites and the diffusion of oxygen molecule during the electrochemical test. Transmission electron microscopy (TEM) images (Fig. 1c, d) also confirm that a large number of micro/macro-pores and amorphous carbon structures can be observed in KB350Z-900. Significantly, defective and exposed edges in the carbon nanostructure owing to a higher percentage of N-doping are formed, which is also supposed to offer effectively reactive sites for the ORR [15].

**Fig. 2:**
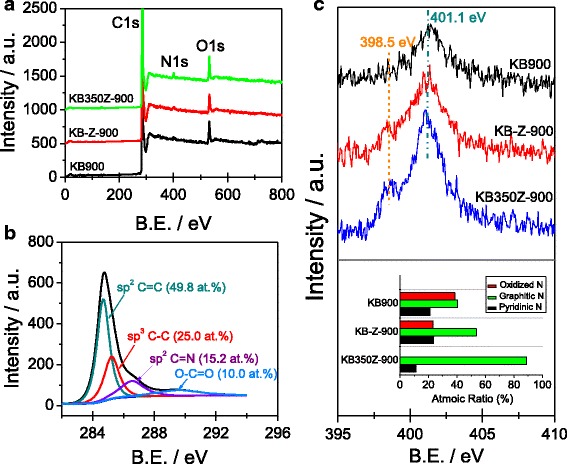
**a** XPS survey of KB900, KB-Z-900 and KB350Z-900; **b** C1s XPS spectrum of KB350Z-900; **c** N1 s XPS spectra of KB900, KB-Z-900 and KB350Z-900

Figure 2a, b shows that the nitrogen atoms are successfully doped into the carbon structure of three types of ORR catalysts. The surface nitrogen content from XPS analysis is 1.23, 1.92, and 2.70 at.% for KB-900, KB-Z-900, and KB350Z-900, respectively. It indicates that the nitrogen loss can be decreased owing to the activation of ZnCl_2_ and two-step carbonization process [6]. The N1 s XPS spectra of KB-900 and KB-Z-900 can be fitted to three peaks (see Additional file 1: Figure S2), which can be ascribed to pyridinic-N, graphitic-N and oxidized-N [6–8, 12, 13], respectively. However, the N1 s XPS spectrum of KB350Z-900 can be fitted into only two peaks (see Additional file 1: Figure S2), centered at 398.5 and 401.1 eV, which can be assigned to pyridinic-N and graphitic-N, respectively. Notably, the oxidized-N species is not observed at N1 s XPS spectrum of KB350Z-900, and the percentage of graphitic-N species is up to 88.8 at.% in total nitrogen content. The content of graphitic-N species follows the order of KB350Z-900 > KB-Z-900 > KB-900, implying that the ZnCl_2_ activation process can be easy to facilitate the increase of graphitic-N content inside the NC material and the usage of KB350 precursor derived from the fist-step pretreatment of KB material can effectively reduce the formation of oxidized-N species.

**Fig. 3:**
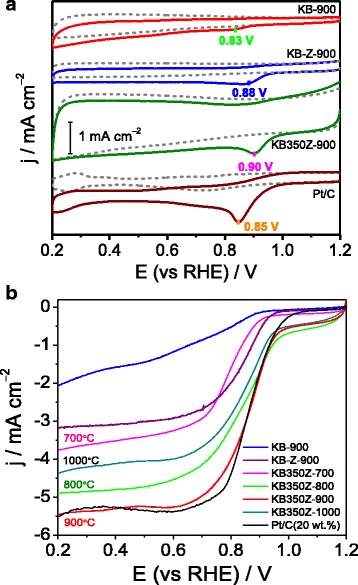
**a** CV curves of KB900, KB-Z-900 and KB350Z-900 in N_2_ versus O_2_ saturated KOH solution; **b** LSV curves of the prepared catalysts and JM Pt/C catalyst

CV curves (see Fig. 3a) obtained in N_2_ versus O_2_ saturated KOH solutions demonstrate that the KB350Z-900 exhibits the highest ORR peak current density and the most positive peak potential (0.90 V) compared to KB-Z-900 and KB-900, which can be owing to high content of graphitic-N species insides the catalyst [13, 16]. In addition, LSV curves (Fig. 3b) obtained in O_2_ saturated KOH solution further indicate that the ORR half-wave potential and limited current density of KB350Z-900 approach to those of the first-class 20 wt.% Pt/C catalyst. The Tafel method was used to analyze the currentepotential (j-E) curves in the kinetic range. The ORR current density is nearly independent of the electrode rotation rate in the potential range of 0.8–1.0 V (vs. RHE), suggesting that the current density in this low-overpotential range is dominated by the electrochemical kinetic current density. The Tafel plots of E as a function of log (j) are shown in (Additional file 1: Figure S3). A Tafel slope of 143 mV decade^− 1^ is obtained for KB350Z-900. The deviation of the Tafel slopes for both KB350Z-900 and Pt/C catalyst implies that their intermediate adsorption may follow a different model [17]. Higher Tafel slopes (absolute value) correspond to a rapid increase in overpotential with current density, probably leading to a relatively inferior ORR catalytic activity [18]. However, the ORR electrocatalytic activity of KB350Z-900 can be more excellent compared to the previously reported carbon-based catalysts derived from other biomass or biomaterial [9–14]. The study of pyrolysis temperature effect on NC catalysts via the ZnCl_2_ activation also displays that the ORR activity follows the order of KB350Z-900 > KB350Z-800 > KB350Z-1000 > KB350Z-700, suggesting the best electrocatalytic activity of KB350Z-900, as higher or lower temperatures will cause the ORR activity to be worse in alkaline medium [19]. It may be mainly attributed to a valuable reason that high porosity and specific surface area, and high surface N content, and percentage of N species of KB350Z-900 can facilitate the fast transportation of O_2_ molecule and the exposure of accessible active sites [6], which can help to enhance the electrocatalytic activity towards the ORR.

**Fig. 4:**
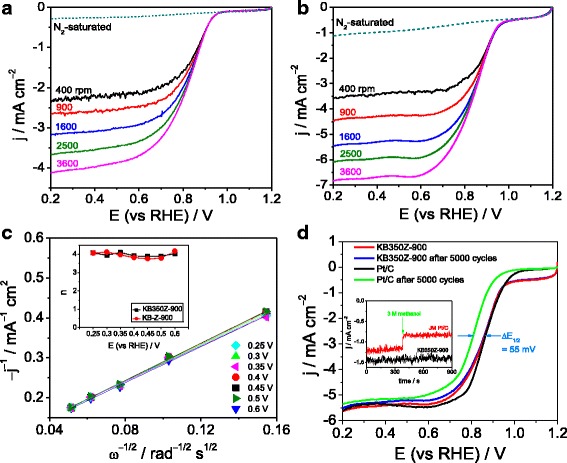
**a** LSV curves of KB-Z-900 in N_2_ versus O_2_ saturated KOH solution at different rotation rates; **b** LSV curves of KB350Z-900 in N_2_ versus O_2_ saturated KOH solution at different rotation rates; **c** Koutecky-Levich plots of j_d_^− 1^ versus ω^− 1/2^ obtained from (**b**) at the given potentials (0.25–0.6 V). Inset is the plots of n versus potential obtained from (**a**) and (**b**); **d** LSV curves of KB350Z-900 and JM Pt/C before and after CV for 5000 cycles in O_2_ saturated KOH solution; Inset is amperometric i-t curves at 0.9 V versus RHE for methanol-tolerant testing

Furthermore, LSV curves for ORR of KB-Z-900 and KB350Z-900 at different rotation rates (400–3600 rpm) are shown in Fig. 4a, b. Good linearity of Koutecky–Levich plots (Fig. 4c) indicates the first-order ORR kinetics with regard to dissolved-O_2_ concentration. The average electron transfer number (n) of the ORR on the KB-Z-900 and KB350Z-900 is estimated to be ~ 3.93 and ~ 3.98 (inset of Fig. 4c), respectively, according to the Koutecky-Levich equation [20]. The Koutecky-Levich equation is as follows:

$$ 1/{j}_d=1/{j}_k+1/B{\omega}^{1/2} $$

$$ \mathrm{B}=0.62\mathrm{nF}{\mathrm{C}}_{\mathrm{O}}{\mathrm{D}}_{\mathrm{O}}^{2/3}{\nu}^{-1/6}{\upomega}^{1/2} $$

where *F* is the Faraday constant, *C*_O_ is the O_2_ saturation concentration in the electrolyte, *D*_O_ is the O_2_ diffusion coefficient in the electrolyte, *ν* is the kinetic viscosity of the electrolyte, and *ω* is the electrode rotation speed, and 0.62 is a constant when the rotation rate is expressed in rpm. It suggests the ORR process on KB-Z-900 and KB350Z-900 mainly follows a direct four-electron transfer pathway to produce H_2_O (e.g., O_2_ + 2 H_2_O + 4e^−^ → 4 OH^−^), which is very similar to the ORR catalyzed by a Pt/C catalyst [21].

Here, we use the accelerated aging test (AAT) by CV continuous scanning for 5000 cycles on a potential range of 0.2 to 1.2 V versus RHE to evaluate the electrochemical stability of KB350Z-900 and Pt/C catalyst in an O_2_-saturated KOH electrolyte. After CV testing, the half-wave potential of the ORR on the KB350Z-900-catalyzed electrode is negatively shifted by only 2 mV, but the reduced half-wave potential of the ORR on the JM Pt/C-catalyzed electrode is about 55 mV (Fig. 4d). Additionally, a higher degradation in limited current density is also found for Pt/C catalyst, indicating more excellent electrocatalytic stability of KB350Z-900 towards the ORR. Amperometric i-t curves at 0.9 V in O_2_-saturated KOH electrolyte (inset of Fig. 4d) confirm that the electro-oxidation reaction of 3 M methanol hardly occurs at KB350Z-900, suggesting a good methanol-tolerant performance of KB350Z-900 and the promising applications in alkaline methanol fuel cells.

## Conclusions

Herein, we develop a facile and easy method to the large-scale production of high-porosity carbons doped with heavily graphitic nitrogen from two-step pyrolysis of kidney bean biomass combining with the activation of zinc chloride and acid-treatment process, which can be functioned as an oxygen reduction electrocatalyst in alkaline medium. First, we find that a large BET surface area (~ 1132 m^2^ g^− 1^) can be obtained at KB350Z-900 with a high pore volume of ~ 0.62 m^3^ g^− 1^. Secondly, two-step pyrolysis process with zinc chloride activation can help to significantly increase the content of graphitic nitrogen inside the carbon-based catalyst. We also observe that the ORR catalytic activity of this carbon material can compare favorably with that of the state-of-the-art commercial 20 wt.% Pt/C catalyst, but also the former’s electrocatalysis stability to the ORR and methanol-tolerant performance are better, suggesting a promising applications in alkaline fuel cells. The excellent ORR performance of KB350Z-900 can be mainly owing to high content of graphitic nitrogen, high specific surface area, and porous characteristics. Our results can further promote the large-scale production of highly active and stable carbon-based ORR electrocatalysts derived from widely-existed natural biomass.

## Additional file

Additional file 1: Supporting Information. (DOCX 156 kb)
