# Supplementary material for: Heavily Graphitic-Nitrogen Self-doped High-porosity Carbon for the Electrocatalysis of Oxygen Reduction Reaction
Source: Nanoscale Res Lett. 2017 Nov 17;12:595. doi: 10.1186/s11671-017-2364-6 (PMC5691822; doi:10.1186/s11671-017-2364-6)
Supplement: Additional file 1: — Supporting Information. (DOCX 156 kb) [file 11671_2017_2364_MOESM1_ESM.docx]

**Supporting Information**

Heavily Graphitic-Nitrogen Self-doped High-porosity Carbon for the Electrocatalysis of Oxygen Reduction Reaction

Tong Feng^a†^, Wenli Liao^a†^, Zhongbin Li^a†^, Lingtao Sun^a^, Dongping Shi^a^, Chaozhong Guo^a*^, Yu Huang^a^, Yi Wang^a^, Jing Cheng^a^, Yanrong Li^a^, Qizhi Diao^b*^

^a^ Research Institute for New Materials Technology, School of Chemistry and Chemical Engineering, Engineering Research Center of New Energy Storage Devices and Applications, Chongqing University of Arts and Sciences, Chongqing 402160, China.

^b^ Central Laboratory Yongchuan Hospital, Chongqing Medical University, Chongqing 402160, China

†These authors equally contributed to this work, and they are considered as co-first author.

**E-mail:** [2945194383@qq.com](mailto:2945194383@qq.com) (Tong Feng); [liaowenli@cqwu.edu.cn](mailto:liaowenli@cqwu.edu.cn) (Wenli Liao); [lzb@cqwu.net](mailto:lzb@cqwu.net) (Zhongbin Li); [ltsun@cqwu.net](mailto:ltsun@cqwu.net) (Lingtao Sun); [7524497@qq.com](mailto:7524497@qq.com) (Dongping Shi); [guochaozhong1987@163.com](mailto:guochaozhong1987@163.com) (Chaozhong Guo); [876988406@qq.com](mailto:876988406@qq.com) (Yu Huang); [489525742@qq.com](mailto:489525742@qq.com) (Yi Wang); [894882629@qq.com](mailto:894882629@qq.com) (Jing Cheng); [1793474272@qq.com](mailto:1793474272@qq.com) (Yanrong Li); [diaoqizhi@163.com](mailto:diaoqizhi@163.com) (Qizhi Diao)

***Corresponding authors.** E-mail: guochaozhong1987@163.com (C. Guo)

Figure S1. TG/DTG curves of white KB biomass.

Figure S2. XPS spectra for N1s region of KB-Z-900 (a) and KB350Z-900 (b).

Figure S3. Tafel plots for ORR on KB-900, KB-Z-900, KB350Z-900.
